# Characterization of a murine model of endothelial dysfunction induced by chronic intraperitoneal administration of angiotensin II

**DOI:** 10.1038/s41598-021-00676-x

**Published:** 2021-10-27

**Authors:** Celeste Trejo-Moreno, Enrique Jiménez-Ferrer, Gabriela Castro-Martínez, Marisol Méndez-Martínez, María Angélica Santana, Gerardo Arrellín-Rosas, José Pedraza-Chaverri, Omar Noel Medina-Campos, Beatriz Hernández-Téllez, Oscar Ramírez-Pliego, Maribel Herrera-Ruiz, Jacquelynne Cervantes-Torres, Zimri Aziel Alvarado-Ojeda, Alejandro Costet-Mejía, Gladis Fragoso, Gabriela Rosas-Salgado

**Affiliations:** 1 Facultad de Medicina, Universidad Autónoma del Estado de Morelos, 62350 Cuernavaca, Morelos Mexico; 2 Centro de Investigación Biomédica del Sur, Instituto Mexicano del Seguro Social, 62790 Xochitepec, Morelos Mexico; 3 Departamento de Sistemas Biológicos, Universidad Autónoma Metropolitana-Xochimilco, 04960 Mexico City, Mexico; 4 Centro de Investigación en Dinámica Celular, Universidad Autónoma del Estado de Morelos, 62209 Cuernavaca, Morelos Mexico; 5 Facultad de Ciencias de la Salud, Universidad Panamericana, 03920 Mexico City, Mexico; 6 Facultad de Química, Universidad Nacional Autónoma de México, 04510 Mexico City, Mexico; 7 Facultad de Medicina, Universidad Nacional Autónoma de México, 04510 Mexico City, CP Mexico; 8 Instituto de Investigaciones Biomédicas, Universidad Nacional Autónoma de México, 04510 Mexico City, Mexico; 9 Posgrado en Biología Experimental, Universidad Autónoma Metropolitana-Iztapalapa, 09640 Mexico City, Mexico

**Keywords:** Cell biology, Immunology, Biomarkers

## Abstract

Endothelial dysfunction (ED) is a key factor for the development of cardiovascular diseases. Due to its chronic, life-threatening nature, ED only can be studied experimentally in animal models. Therefore, this work was aimed to characterize a murine model of ED induced by a daily intraperitoneal administration of angiotensin II (AGII) for 10 weeks. Oxidative stress, inflammation, vascular remodeling, hypertension, and damage to various target organs were evaluated in treated animals. The results indicated that a chronic intraperitoneal administration of AGII increases the production of systemic soluble VCAM, ROS and ICAM-1 expression, and the production of TNFα, IL1β, IL17A, IL4, TGFβ, and IL10 in the kidney, as well as blood pressure levels; it also promotes vascular remodeling and induces non-alcoholic fatty liver disease, glomerulosclerosis, and proliferative retinopathy. Therefore, the model herein proposed can be a representative model for ED; additionally, it is easy to implement, safe, rapid, and inexpensive.

## Introduction

The endothelium is a single-cell lining that covers the internal surface of blood vessels, cardiac valves, and various body cavities^1,2^. It acts as a gatekeeper, sensing and responding to stimuli (physical or chemical, like changes in blood flow or pressure, inflammatory signals, or the levels of circulating hormones) and activating vasoactive systems that help maintaining vasomotor balance and homeostasis in vascular tissues. The endothelium produces both agonistic and antagonistic molecules that help keeping homeostasis^2^. When this balance is disrupted, it favors vasculature vasoconstriction, leukocyte adherence, platelet activation, mitogenesis, pro-oxidation, thrombosis, impaired coagulation, vascular inflammation, and atherosclerosis. This altered condition is known as endothelial dysfunction (ED), since it reduces the capacity of endothelium to maintain homeostasis and leads to the development of vascular diseases like systemic arterial hypertension (SAHT), renal dysfunction, and cerebrovascular diseases, which are the main causes of morbidity and mortality worldwide^1,3–5^. Angiotensin II (AGII), part of the renin–angiotensin–aldosterone system (RAAS), is actively involved in the pathophysiology of cardiovascular and renal diseases^2,4^. It may be responsible for triggering ED and vascular inflammation by inducing oxidative stress, which in turn causes the upregulation of inflammatory mediators and cell growth factors^4,6–8^. The severity of this pathology and its consequences make necessary to find a suitable animal model to study the pathophysiology of ED and the action mechanisms of the drugs intended to control it^9^.

The administration of exogenous AGII to induce hypertension or study its effects has been widely used, delivered to mice either through an osmotic pump^10^ or by an AGII-coated pellet^11^ implanted under the skin. The model herein presented is a cost-effective variant of this endocrine model of hypertension pharmacologically induced by a chronic administration of AGII^12^ since instead of infusion via a pump or pellet, the hormone was administered by a daily intraperitoneal injection. This method is easy, safe, rapid, and inexpensive.

The model herein proposed is based on 1) an acute hypertension model developed by Jiménez-Ferrer et al.^13^; in that model, the authors established that an i.v. dose of 1 μg/kg of AGII (Sigma) increased SBP by 20% and DBP by about 60% with respect to normal values (120/70 mmHg); and 2) a work performed on isolated, endothelial cell-denuded aorta rings in an organ bath. In this case, an AGII concentration of 10^−6^ M increased a basal tension value of 2 g with respect to a control^14^. With that background, the authors proposed this model, in which subject mice not only showed hypertension, but a complete ED condition, as evinced by prooxidant and proinflammatory activity, vascular remodeling, and damage to target organs.

## Results

### Chronic i.p. administration of AGII induces endothelial dysfunction by increasing of soluble VCAM

**Figure 1:**
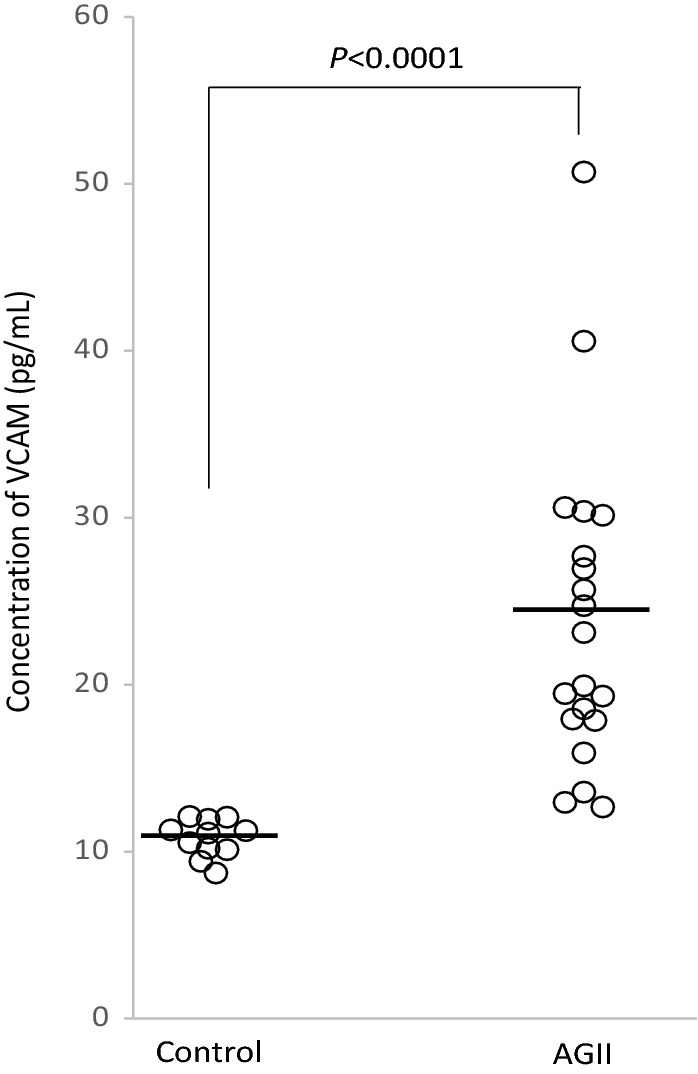
Plasma VCAM levels in AGII-treated mice and controls. The concentration of soluble VCAM was measured in mice treated intraperitoneally with AGII (*n* = 20) and in control, untreated mice (*n* = 11). Bars indicate the mean value in each group. *P* < 0.0001 (Mann–Whitney U-test).

It has been documented that the levels of both endothelium-bound and soluble adhesion molecules increase in endothelial dysfunction^15^. To confirm this in our model, soluble levels of VCAM were measured in plasma samples from AGII-treated mice and from control animals. As shown in Fig. 1, notably increased VCAM levels were found in plasma from AGII-treated mice with respect to untreated animals (24.5 vs. 10.8 pg/mL, respectively).

### Chronic i.p. administration of AGII induces a prooxidant condition by increasing NOX2 and NOX4 transcription

**Figure 2:**
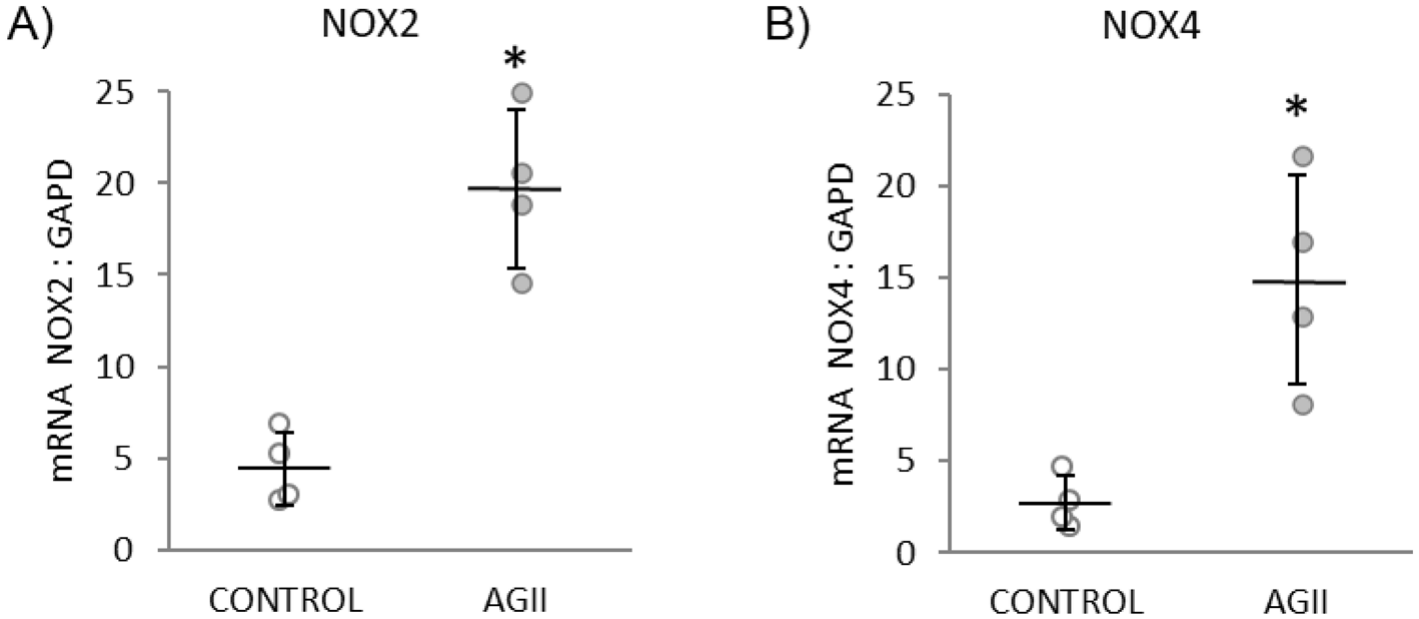
Chronic AGII administration increases the expression of NOX2 and NOX4 mRNA in kidney. Data are reported as mean ± SD (*n* = 4 for each group) and analyzed with the Mann–Whitney U-test (*P* < 0.05); *Indicates differences with respect to controls.

Oxidative stress plays an important role in the pathophysiology of ED and associated cardiovascular diseases (CVDs), like SAHT, atherosclerosis, diabetes, cardiac hypertrophy, heart failure, and ischemia–reperfusion^16^. NADPH oxidases seem to be especially important for redox signaling, and this protein family could be a specific therapeutic target^16^. Six homologous forms of NADPH oxidases constitute the NOX family, which shares the capacity to transport electrons across the plasma membrane to generate superoxide and other downstream ROS. From this family, NOX2 and NOX4 are expressed in the endothelium, and AGII has been reported to induce their transcription. To analyze the expression of the NOX2 and NOX4 homologues in kidney, a real-time quantitative PCR assay was performed, with specific primers for NOX2 and NOX4 mRNAs. Primer specificity was confirmed by PCR amplification of the cloned cDNAs, where no cross-hybridization to other NOX cDNAs was observed (data not shown). The results of PCR analysis are shown in Fig. 2. The expression of NOX2 (Fig. 2A) and NOX4 (Fig. 2B) mRNA showed a 4- and fivefold increase, respectively, in the kidneys of AGII-treated mice with respect to control animals (*P* < 0.05).

### AGII increased eNOS transcription levels

Both eNOS and iNOS produce nitric oxide (NO), but under different conditions. The former is an endothelial constitutive enzyme, and in this case, NO helps maintaining tissue homeostasis. The latter enzyme produces NO as a response to the activation of pro-inflammatory cells after damage or inflammation^17^. AGII has been reported to influence the expression of all three NO synthase (NOS) isoforms in the long term. To determine whose NOS isoform expression was modified by AGII, the expression of both eNOS and iNOS mRNA in mouse kidney was quantified by RT-PCR. After AGII administration, eNOS expression (Supplementary Fig. S1A online) showed a 2.5-fold increase (*P* < 0.05) with respect to the control group. iNOS expression also increased, but not significantly (see Supplementary Fig. S1B online).

### The main ROS source is xanthine oxidase

**Figure 3:**
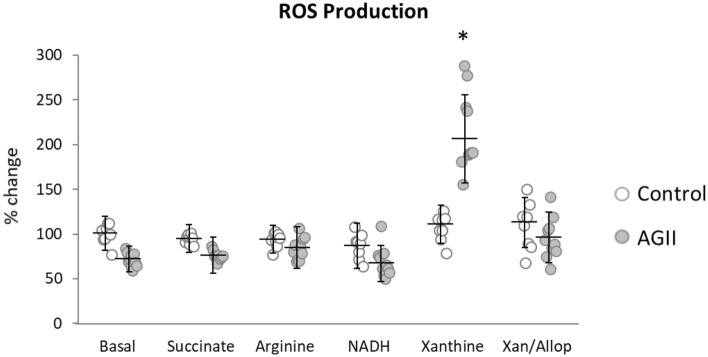
ROS production. AGII failed to induce changes in ROS production when NADH, arginine, or succinate were used as enzyme substrates, while using xanthine as a substrate increased significantly the xanthine oxidase production of ROS; this increase was prevented by incubation with the inhibitor allopurinol. Xan (Xanthine); Allop (Allopurinol). Eight kidney specimens were analyzed per treatment. Data are reported as mean ± SD and analyzed with the Mann–Whitney U-test (*P* < 0.05); *Indicate differences with respect to controls.

Oxidative stress is a key trait of ED. To determine whether ROS were produced in this model, and which enzyme is responsible for its production, various enzymes reported as ROS producers were evaluated in kidney extracts by quantitative DHE oxidation. Protein extracts from control or AGII-treated mice were exposed to a set of substrates before measuring ROS production (Fig. 3). ROS production increased almost 2 times in AGII-treated animals with respect to controls when the extracts were exposed to xanthine (*P* < 0.01). To confirm this result, the extract was incubated with xanthine and its competitive inhibitor allopurinol, which reduced ROS production. No differences were observed when the extracts were incubated with NADH, arginine, nor succinate. These data suggest that xanthine oxidase is involved in ROS production in response to chronic AGII administration, as expected in an ED model.

### Chronic i.p. AGII administration induces a proinflammatory condition

**Figure 4:**
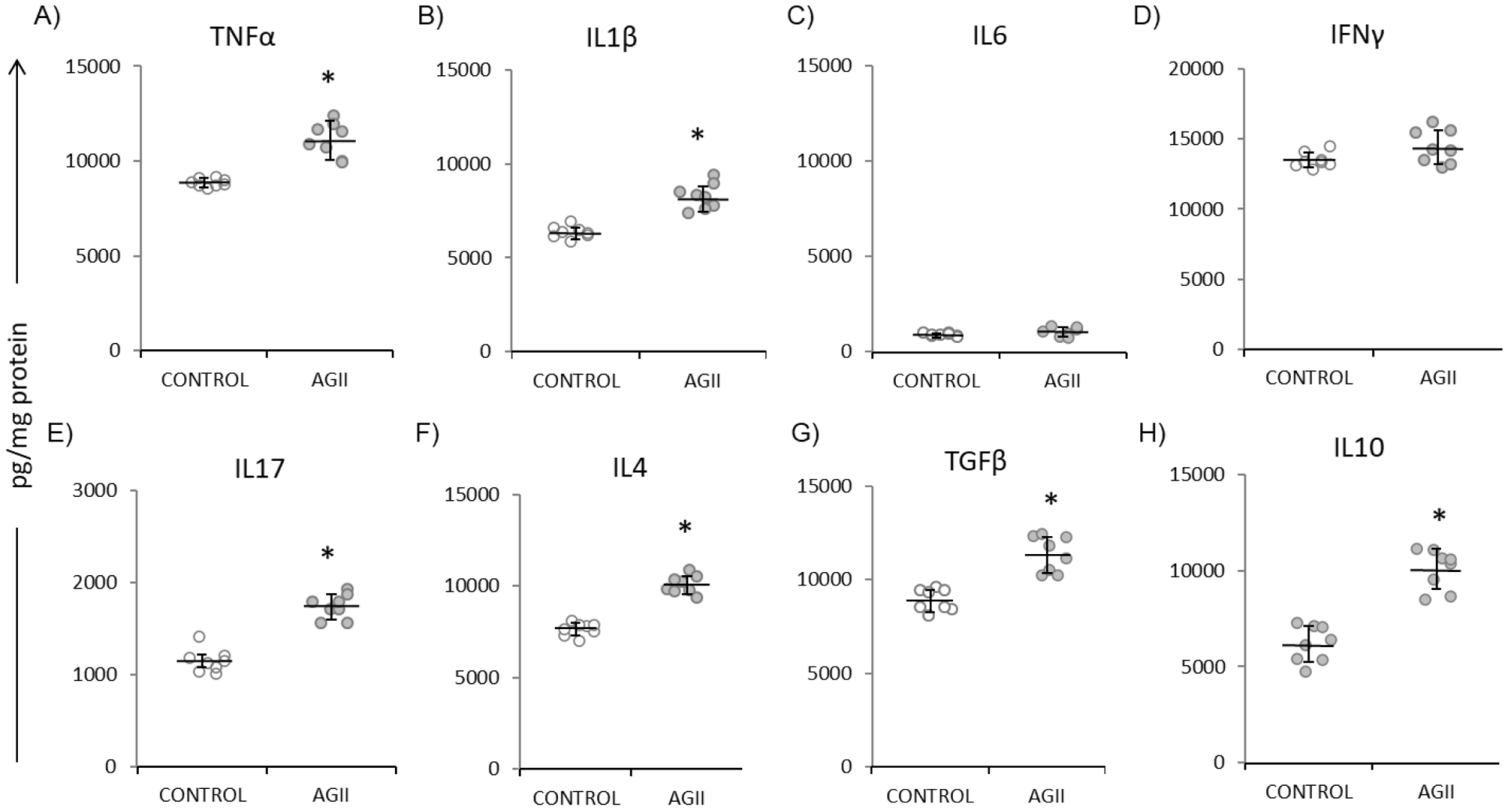
Cytokine concentrations in kidney. AGII administration for 10 weeks increased cytokine levels (**A**–**H**), both proinflammatory (**A**,**B**,**E**) and regulatory (**G**–**H**). Eight kidney specimens were analyzed per treatment. Data are reported as mean ± SD and analyzed with the Mann–Whitney U-test (*P* < 0.05); *Indicate differences with respect to controls.

Low-grade inflammation in the endothelium is a major contributor to the development of CVD^18^. It has been reported that CVD patients show increased plasma levels of cytokines like the tumor necrosis factor-alpha (TNFα) and interleukin 6 (IL6), as well as adhesion molecules like the intercellular adhesion molecule-1 (ICAM-1), the vascular cell adhesion molecule-1 (VCAM-1), and E-selectin, among other inflammatory markers^2^. Thus, inflammation, a central mechanism in the progression of ED and CVD^3^, was also evaluated in this model. Pro-inflammatory status was assessed in mouse kidney samples obtained after the last BP measure. Cytokine concentrations were determined by ELISA in tissue homogenates; ICAM-1 was detected by immunohistochemistry, and ICAM-1 mRNA was quantified by RT-PCR. As shown in Fig. 4, AGII-treated mice showed significantly increased TNFα (25.2%, Fig. 4A), IL1β (31.1%, Fig. 4B), IL17A (51.3%, Fig. 4E), IL4 (31.7%, Fig. 4F), TGFβ (27.4%, Fig. 4G), and IL10 (64.3%, Fig. 4H) levels with respect to untreated controls, while no differences were observed in IL6 (Fig. 4C) or IFNγ levels (Fig. 4D).

On the other hand, a sixfold increase was observed in the expression of ICAM-1 mRNA in AGII-treated animals with respect to controls (*P* < 0.05, Supplementary Fig. S2G online). ICAM-1 was detected in perirenal fat tissue (Fig. S2D), renal capsule cells (Fig. S2E), and renal interstice cells (Fig. S2F), accompanied by an inflammatory cell infiltrate in renal capsule tissue (Fig. S2E), in the group treated with AGII, while in the control group (Fig. S2A–C) it was not detected. These results indicate that i.p. AGII administration induces an inflammatory condition, as expected in ED.

### Chronic AGII administration leads to hypertension and vascular remodeling

Hypertension induction is one of the most important parameters expected in this model, because it is the most common clinical parameter of ED. AGII is actively involved in the pathophysiology of SAHT, since it triggers increased ROS production by endothelial cells^19^. By reducing NO bioavailability, higher ROS levels increase BP through a two-way mechanism: by altering the inner and outer diameter of blood vessels, thus reducing their lumen (vascular remodeling)^20^; and by affecting the capacity of blood vessels to dilate as normal^17^. Acting together, both effects result in hypertension.

**Figure 5:**
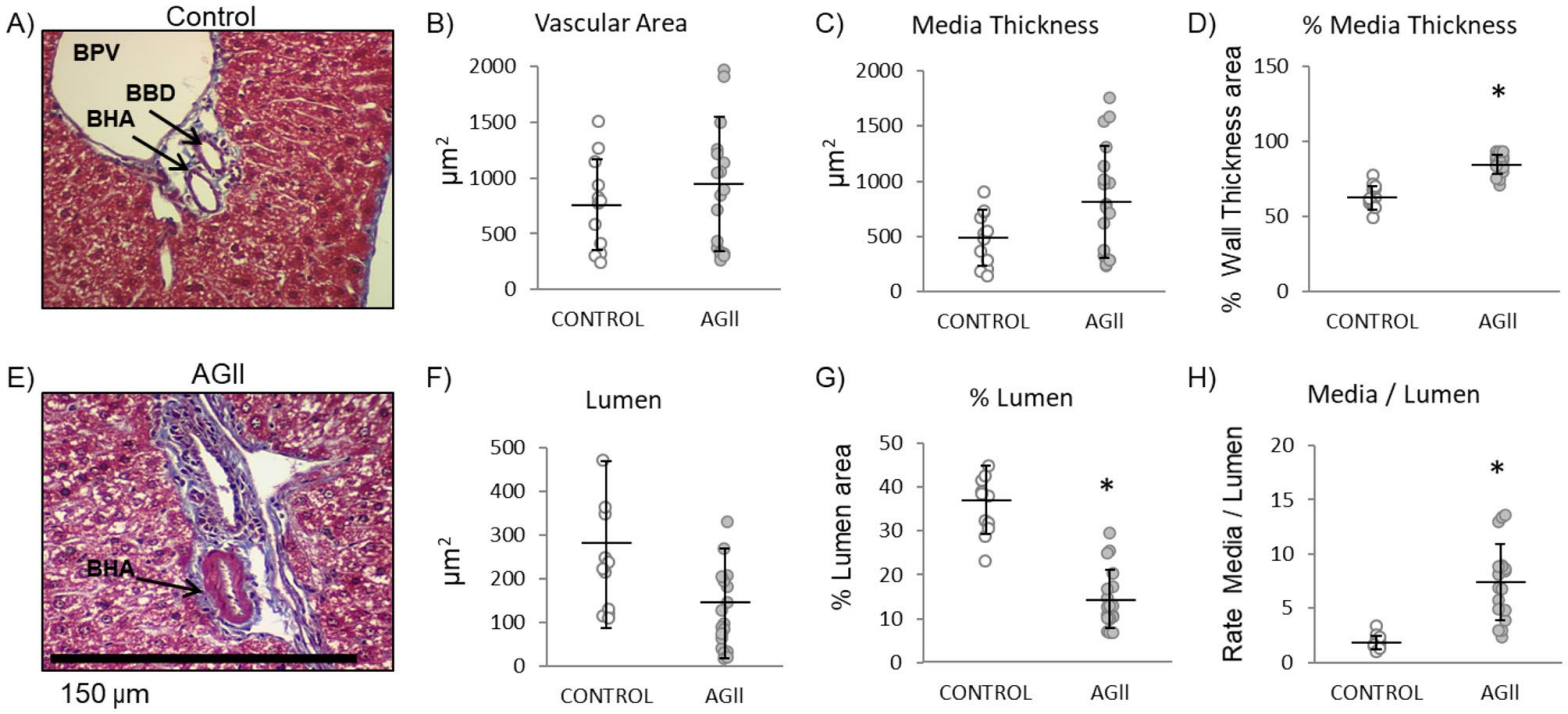
Evaluation of vascular remodeling in BHA by Masson trichrome staining. (**A**,**B**) Hepatic portal triad from treated and control mice. (**C**) Total BHA area. (**D**) BHA lumen area. (**E**) BHA medial thickness. (**F**) Lumen and media (**G**) area percentage with respect to total BHA area. (**H**) Media area/lumen area ratio. BHA thickening is a sign of vascular remodeling; BHA lumen measure was decreased in AGII-treated mice, while medial thickness and media/lumen ratio were increased. Microphotographs taken at 40X. (BPV) Branch of portal vein; (BHA) Branch of hepatic artery; (BBD) Branch of biliary duct. Three kidney specimens were analyzed per group (*n* = 12 BHA samples from control animals and *n* = 19 BHA samples from the AGII-treated group). Data are reported as mean ± SD and analyzed with the Mann–Whitney U-test (*P* < 0.05); * indicate differences with respect to controls.

The measurements and calculations performed in histological slides of the BHA of the portal triad in mouse liver are shown in Fig. 5 (panels A, E). Vascular remodeling was assessed by determining medial thickness, lumen percentage, and the media/lumen ratio (Fig. 5B–D, Fig. 5F–H). Medial thickness was calculated by subtracting the luminal BHA area from the total BHA area. As shown in Fig. 5, neither the total BHA vascular area, medial thickness, nor lumen area (panels B, C, and F respectively) were affected by AGII administration, but a twofold decrease was observed in lumen percentage with respect to control mice (*P* < 0.05, Fig. 5G). Medial thickness (Fig. 5D) and the media/lumen ratio (Fig. 5H) showed a 1.3- and 3.7-fold increase, respectively, which indicate hypertrophic vascular remodeling^18^. These results suggest that chronic AGII administration induces vascular remodeling, a key part of ED, hindering the flow of blood through the vessels and thus promoting hypertension.

**Figure 6:**
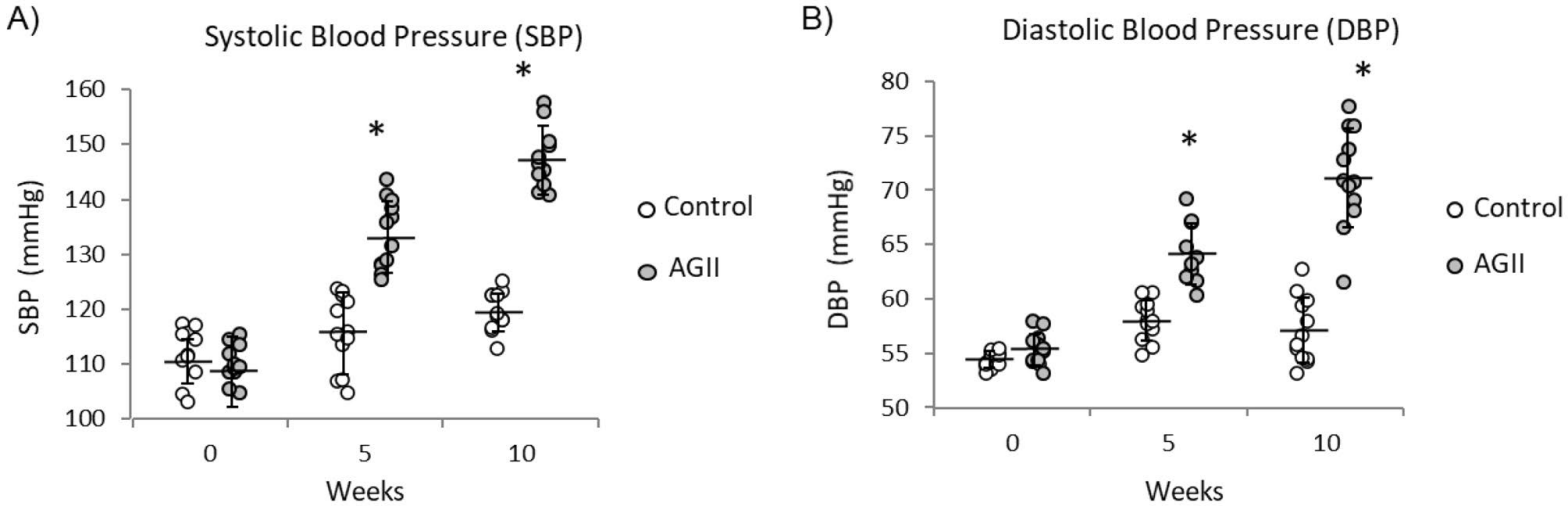
Time-course plot for blood pressure. (**A**) Systolic blood pressure (SBP). (**B**) Diastolic blood pressure (DBP). Blood pressure was measured in mice (*n* = 12 per group) on weeks 0, 5, and 10. Both blood pressure values were increased in AGII-treated mice on weeks 5 and 10, and the animals were regarded as hypertensive. Data are expressed as mean ± SD and were analyzed by ANOVA-Tukey test (*P* < 0.05). *Indicate differences with respect to control.

A time-course plot of BP values in control and AGII-treated mice is shown in Fig. 6. BP was measured at the beginning (week 0), the middle (week 5), and the end of experiment (week 10). A gradual increase of systolic BP (SBP, Fig. 6A) and diastolic BP (DBP, Fig. 6B) was observed in AGII-treated mice. On week 5, SBP and DBP increased by 15% (Fig. 6A) and 16% (Fig. 6B), respectively, with respect to control mice (*P* < 0.05). On week 10, SBP increased by 25% (Fig. 6A) and DBP increased by 24% (Fig. 6B, *P* < 0.05) with respect to control. These results indicate that this model is suitable to study SAHT and its causes, including vascular remodeling, and it also points to the effect of AGII on NO-dependent vascular function.

### Chronic AGII administration provides a promising model of non-alcoholic fat liver disease, glomerulosclerosis, and proliferative retinopathy

Historically, AGII has been regarded as a primary factor of tissue damage^21^ in different organs: it increases ROS production; it also promotes upregulation in the expression of cytokines, cell adhesion molecules, and profibrotic factors like the transforming growth factor-β (TGFβ), which increases the synthesis of extracellular matrix proteins and promote macrophage activation and infiltration^22^, among other harmful responses. AGII treatment induced kidney damage resembling glomerulosclerosis^23^, hepatic damage similar to non-alcoholic steatohepatitis (NASH)^24^, and eye damage resembling proliferative retinopathy^25^.

**Figure 7:**
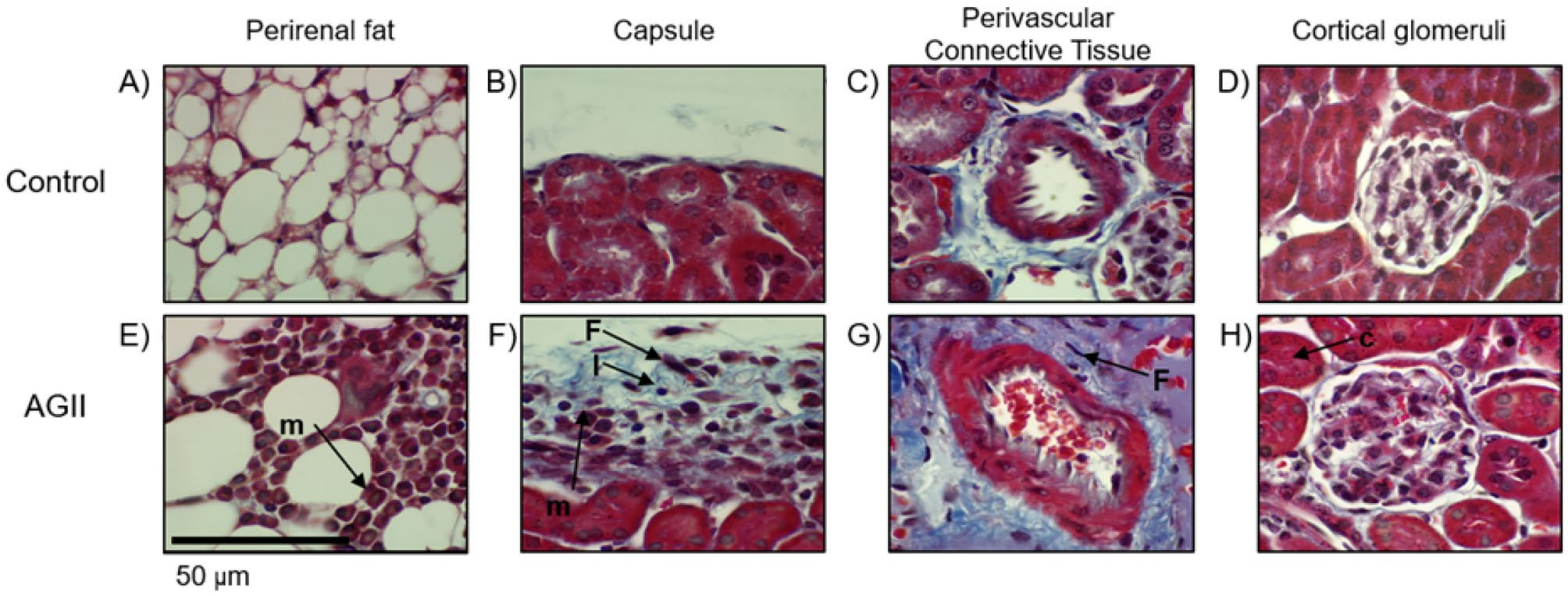
Evaluation of renal damage by Masson trichrome staining. (**A**,**E**) Perirenal fat tissue. (**B**,**F**) Renal capsule. (**C**,**G**) Perivascular connective tissue. (**D**,**H**) Cortical glomeruli. Renal damage was observed in mice treated with AGII for 10 weeks, as evinced by infiltration of inflammatory mononuclear cells and fibroblasts, edema y and fiber deposition. Three kidney specimens were analyzed per group. m: mononuclear cells; (**F**) fibroblasts; l: lymphocyte; c: congestion. Microphotographs taken at 100X.

Glomerulosclerosis is a condition frequently resulting from ED; thus, AGII-induced kidney damage was assessed on Masson trichrome-stained perirenal fat tissue, capsules, perivascular connective tissue, and cortical glomeruli. The type of cells infiltrating the capsule was determined by PAS staining; finally, and a morphometric analysis of glomeruli was performed. As shown in Fig. 7, AGII-treated mice exhibited signs of renal damage, including renal capsule thickening (Fig. 7F) due to edema, fiber deposition, and infiltration of mononuclear cells, mainly lymphocytes and macrophages, characteristic of CKDs like glomerulosclerosis^26^. Additionally, perivascular fibrosis (Fig. 7G), congestion of the tubulointerstitial zone (Fig. 7H) and points of mononuclear infiltration (macrophages and lymphocytes) among perirenal fat adipocytes (Fig. 7E) were observed, while in the control group these characteristics were not detected (Fig. 7A–D).

In AGII-treated mice, the renal capsule thickening, which may be associated to the infiltration of inflammatory cells, mostly lymphocytes, macrophages, plasmatic cells, and fibroblasts, but also basophiles and neutrophils to a lesser extent (Supplementary Fig. S3B), while these cells were not observed in the control group (Fig. S3A). As shown in Supplementary Fig. S3 online, an increase in glomerular area (37.0%, Fig. S3D–E), glomerulus vascular region hypertrophy (52.1%, Fig. S3F), and increased mesangial area (16.1%, Fig. S3G), all histopathological signs of glomerulosclerosis, were observed in AGII-treated mice compared to the control group (Fig. S3C, E–G).

**Figure 8:**
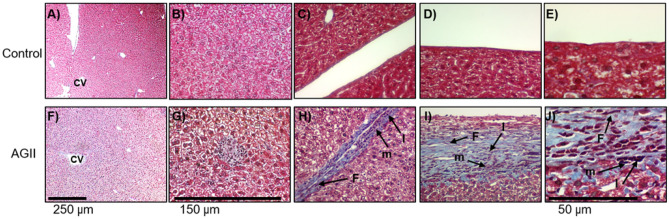
Evaluation of hepatic damage by Masson trichrome staining. (**A**,**B**,**F**,**G**) Hepatic parenchyma. (**C**,**H**) Trabeculae. (**D**,**E**,**I**,**J**) Glisson’s capsule. AGII-treated mice showed steatohepatitis (**F**) and lymphocytic microabscesses (**G**). Additionally, trabeculae and Glisson’s capsule showed mononuclear cell infiltrate and fiber deposition, leading to their thickening (**H**–**J**). Three liver specimens were analyzed per group. CV: centrilobular veins; m: mononuclear inflammatory cells; F: fibroblasts; l: lymphocytes. Microphotographs taken at 10X (bar = 250 μm), 40X (bar = 150 μm) and 100X (bar = 50 μm).

On the other hand, three pathologic liver alterations were found in AGII-treated mice: 1) Steatosis with a perivascular pattern stemming from the central vein of the hepatic lobule (Fig. 8F); 2) thickening of trabeculae (Fig. 8H) and Glisson’s capsule (Fig. 8I–J), and 3) lymphocytic microabscesses with central necrosis (Fig. 8G). All these alterations, steatohepatitis, inflammation, and fibrosis are pathological traits of non-alcoholic steatohepatitis (NASH), while these characteristics were not observed in the control group (Fig. 8A–E). The thickening of trabeculae and Glisson’s capsule is due to fiber deposition and the infiltration of mononuclear cells like lymphocytes, macrophages, fibroblasts, and fibrocytes, which account for fibrosis (Fig. 8H–J), indicate the chronicity of the inflammatory event and the ongoing repairing process.

With respect to the eye, neovascularization is a hallmark of proliferative retinopathy, where the growth of abnormally formed blood vessels leads to hemorrhage, vision loss, and blindness^27^. As shown in Supplementary Fig. S4, no significant differences in the number of blood vessels (Fig. S4A,B) were found between control and AGII-treated mice. However, AGII administration increased the formation of neovessels (*P* < 0.05, Fig. S4C,D).

## Discussion

The implementation of an endothelial dysfunction (ED) model induced by the daily administration of a sub-chronic dose of AGII is reported herein. The model performance was confirmed by increased levels of the soluble adhesion molecule, sVCAM, a parameter known to increase during this pathology^15^. ED is characterized by alterations in endothelium activity in which oxidative stress and inflammation act simultaneously, leading to vascular remodeling, limited vasorelaxation, and fibrosis; in turn, these signs are linked to CVDs like hypertension, coronary artery disease, chronic heart failure, peripheral artery disease, cerebral vascular disease, diabetes, and chronic renal failure^4,28^. Interestingly, liver, kidney, and eye damage similar to that reported in non-alcoholic steatohepatitis (NASH), glomerulosclerosis, and retinopathy was observed. The severity of these diseases makes necessary to have a well-characterized animal model that represents ED pathophysiological frame. The factor that triggers the pathophysiological process is the cornerstone of the experimental model. Thus, we proposed an overstimulation of RAAS as the triggering factor for permanent endothelial damage, and a chronic administration of sub-effective doses of AGII was the factor chosen to accomplish this. AGII has been used to induce hypertension in various animal models for ED. The main differences between those models and the one herein reported is the route for AGII administration and the dose employed. The main advantages of this model are its low cost and ease of implementation since no additional equipment or materials are required. While the route of administration could indeed be uncomfortable for the mouse, the animals did not show any clinical or behavioral alteration suggestive of a poorer quality of life due to the drug intraperitoneal administration. On the other hand, the model herein described is minimally invasive, with no anesthesia, shaving, nor surgery required for AGII infusion as it is the case with osmotic pump implantation. As reported by Morton et al.^29^, if the inoculum is kept within pH limits (7.4) that do not cause alteration in the serosa of abdominal organs, and adequate volumes are inoculated (10 ml/kg), a daily intraperitoneal administration is safe. Both points were safely met by this model.

In contrast with other models, we applied a dose of 0.1 µg/kg/day in a volume of 250–300 µl at a pH of 7.4 for 10 weeks to induce a condition analogous to the chronic degenerative process and the possible complications in human patients. Other models administer a higher dose within a shorter period; for instance, the dose used in the osmotic pump model ranges from 280 to 3200 µg/kg/day^10,30–32^ depending on the author, with an overall duration of 1–4 weeks^10,31^, while a dose of 6 µg/kg/day was used in the model of AGII-impregnated pellets for 21–60 days^11^. Despite the variable doses and induction times, most models coincide in that systolic blood pressure values increased by about 40 mmHg^10,11^, as in our model. In addition to this parameter, we also showed that sVCAM, NOX2, NOX4, and iNOS expression, as well as ROS production, vascular remodeling, cellular infiltration, fibrosis, mesangial expansion, and liver inflammation increased after AGII intraperitoneal administration, along with the concentration of TNFα, IFNγ, IL1β, VCAM-1, ICAM-1 and TGFβ.

Some of these parameters have also been reported as increased in aorta^10,32,33^ samples, peripheral blood cells^10^, heart^11^, kidney^31^, or liver tissues^34^ in osmotic pump- or pellet-based models. This suggests that the condition observed in our model is similar to those found in those reported models, with the advantages of a lower cost and not requiring additional materials. As reported, AGII induced the production of ROS (such as superoxide) through NAD(P)H oxidase, xanthine oxidase, mitochondrial enzymes, and uncoupled NOS. These molecules are responsible for nitric oxide degradation linked to endothelial dysfunction^3,35^. As observed in our study, among these four enzymes, only xanthine oxidase (XO) produced ROS, a result in line with the work by Landmesser^35^, who reported a marked increase in endothelial XO levels and in XO-dependent endothelial superoxide production after AGII administration, suggesting that AGII-activated XO is a major superoxide source in coronary diseases resulting from ED. It is interesting to consider that, although eNOS and NADPH did not participate in ROS generation, a significant increase in mRNA expression for eNOS, NOX2, and NOX4 was observed, probably because superoxide ion is rapidly degraded by superoxide dismutase to produce hydrogen peroxide (H_2_O_2_), as previously reported^36^; H_2_O_2_ is an extremely potent stimulus for the gene expression of eNOS^37^, NOX2 (mediated by JnK), and NOX4 (which proceeds through P38MAPK)^38–40^.

With respect to the inflammatory status observed in our model, AGII is known as a proinflammatory stimulant that attracts immune cells to the vascular wall, enhancing the production of cytokines and adhesion molecules^2,3^. Indeed, in our model, AGII-treated mice showed increased levels of inflammatory cytokines, ICAM-1 expression, and the infiltration of immune cells (macrophages, lymphocytes, plasmatic cells, basophils, and neutrophils) in the kidney, a well-known target organ for ED that plays a crucial role in the pathogenesis of ED and SAHT^5^. Moreover, the combined effect of AGII, oxidative stress, and inflammation induces fibrosis, as well as the proliferation and migration of vascular smooth muscle cells (VSMC), which further contribute to vascular remodeling ang hypertension^3,4^. The observed increase in blood pressure can be explained in part because AGII and ROS activate the ERK1/2^41,42^, JNK and NF-kB signaling pathways^43–45^, leading to the activation of matrix metalloproteinases (MMPs) and the production of growth factors like the epidermal growth factor (EGF), platelet-derived growth factor (PDGF), and insulin-like growth factor (IGF)^4,8^. These molecules promote the accumulation of extracellular matrix proteins (collagen and fibronectin) and the proliferation and migration of VSMC, which result in vascular remodeling^4,18^. While we did not study these signaling pathways, they are expected to be involved because of the high ROS levels observed upon AGII administration. High blood pressure values also depend on the levels of NO, which induces vasorelaxation. In this case, NO will not be available because it can react with ROS to produce molecules like peroxynitrite (ONOO^−^); furthermore, peroxynitrite by itself affects NO production by uncoupling eNOS, so it will stop synthesizing NO, producing more O_2_^−^ instead^46^. In our model, hypertension was induced by vasoconstriction and vascular remodeling, which could be related with a lower NO bioavailability.

The fact that the histopathological damage observed in this model resembled those found in NASH, glomerulonephritis, and human retinopathy, could be one of the most interesting results of our work, since they confirm the link between AGII and ED in the origin and progression of pathological damage. Since all affected organs are highly vascularized^47^, the status of the endothelium is key for the organ health. On the other hand, all three diseases have oxidative stress and inflammation as common traits in their origin and progression^24,25,48^.

The use of the intraperitoneal route to administer AGII implies that the changes observed, besides endothelial dysfunction, could be due to the effect of AGII on peritoneal tissues like the visceral adipose tissue, in which it induces differentiation and dysfunction, favoring thus a prooxidant and proinflammatory status^49^.

Oxidative stress and inflammation, induced by both AGII and ED, promote the progression of liver diseases like NASH^50,51^, whose histopathological characteristics, such as steatosis, cellular infiltrate, and fibrosis, were evident in the liver of AGII-treated mice. This prooxidative and proinflammatory environment induces metabolic changes in hepatocytes; indeed, TNFα^52^ and ROS^50^ induce insulin resistance, favoring lipolysis and increasing the serum levels of free fatty acids. In addition, both TNFα and ROS activate SREP-1, which in turn leads to lipogenesis^50^. On the other hand, AGII itself affects hepatic stellate cells (HSC), Kuffer cells, and hepatocytes^53,54^. The accumulation of lipids and their simultaneous degradation induce an erratic condition in hepatocytes that results in three events: increased ROS levels, cell death by necrosis, and inflammation^24^. The presence of a cellular infiltrate enriched with neutrophils and lymphocytes that accompanied necrosis and inflammation^24^, along with Kuffer cells, which are activated by AGII^54^, was observed in our model, as shown in Fig. 8. Fibrosis, a consequence of chronic liver damage in which extracellular matrix is produced to replace dead cells^53^, was also induced in out model. It has been proposed that fibrosis results from to ED (due to TGFβ activity), while AGII^53^ induces the transformation of HSC into myofibroblasts, which eventually produce liver fibrosis^55^. These results indicate that ED, acting together with AGII, play an important role in the pro-steatotic environment that leads to NASH, further supporting our model of endothelial dysfunction induced by AGII.

Mice treated with AGII in our model also developed glomerulosclerosis. The pro-oxidant, pro-inflammatory and profibrotic environment induced by ED has been reported to induce an excessive accumulation of the extracellular matrix in the glomeruli, along with increased levels of vasoconstrictor substances, and an accumulation of type-I collagen and fibronectin. All these changes damage nephrons and may result in their loss^48^. It is known that AGII increases the synthesis of matrix molecules in the kidney, and it could stimulate macrophage infiltration, which favors the progression of sclerosis and promotes interstitial fibrosis, resembling glomerulosclerosis on histopathological examination^2,3^. The results herein reported are interesting because, while the mechanisms underlying the effects of AGII on renal cells are not fully understood, they highlight the effect of this octapeptide on nephrons, either directly or indirectly.

Finally, the ocular damage observed in our model was similar to that reported for diabetic retinopathy^25^. Diabetic retinopathy is due to a microangiopathy affecting retinal precapillary arterioles, capillaries, and venules. The damage is due to both microvascular leakage from a breakdown of the inner blood-retinal barrier and microvascular occlusion^56^. ED has been reported to contribute to ocular damage by oxidative stress^25^ and the persistent low-grade inflammation that accompanies it^57^; both conditions were found in mice treated intraperitoneally with AGII. On the other hand, the renin-angiotensin system is a causative factor of diabetic microvascular complications, inducing a variety of tissular responses, including vasoconstriction, inflammation, oxidative stress, cell hypertrophy and proliferation, angiogenesis, and fibrosis. All the components of the renin-angiotensin system, including angiotensin type-1 and type-2 receptors, have been identified in the retina of humans and rodents^58^. These results are of interest since, although the origin of this disease is not well understood^57^, our results indicate the relevance of ED and AGII in its establishment, which may be useful to develop novel treatment approaches.

In summary, our results show that chronic administration of AGII by a daily intraperitoneal injection leads to endothelial dysfunction characterized by prooxidant and proinflammatory activity, vascular remodeling, hypertension, and damage to organ targets associated with fibrosis.

## Materials and methods

### Animals and housing

All experiments were performed on 10-weeks-old, male C57BL/6 J mice. The animals were housed in groups of five in standard polypropylene cages. The mice were maintained in a 12:12-h light–dark cycle, fed with standard rodent chow and allowed freshwater ad libitum. All procedures were performed in accordance to the guidelines established by the official Mexican regulation NOM-062-ZOO-1999 (technical specifications for production, care and use of laboratory animals). Experimental protocols were reviewed and approved by the Ethical Committee for the Care and Use of Laboratory Animals (Permit No. 005/2011) at the School of Medicine of Universidad Autónoma del Estado de Morelos (FM-UAEM) and complied with the ARRIVE guidelines (https://arriveguidelines.org).

### Control and experimental group

Each experiment lasted for 10 weeks and included 22 and 15 mice in the experimental and the control group, respectively. The animals in both groups were distributed into five repetitions, four with 4 animals each, and one with 6 for the experimental group, while three control mice were included in each repetition. AGII treatment was applied daily by the intraperitoneal (i.p.) route. Control mice received isotonic saline solution (ISS) only, while the experimental group received 0.1 μg/kg of AGII. It should be noted that not all outcome variables were measured in all repetitions; thus, different repetitions were used to measure different variables.

### Organ retrieving

After the last blood pressure (BP) measurement, the mice were perfused with cold PBS under surgical anesthesia (xylazine, 10 mg/kg, i.p.), and the liver and kidneys were obtained. For histopathological and immunohistochemical studies, the organs were fixed in Zamboni’s solution (2.0% formaldehyde, 0.2% picric acid, pH 7.0). To measure oxidant stress and interleukin levels, the organs were weighed and frozen at − 80 °C until used. For real time PCR assays, the organs were preserved in Trizol reagent (Invitrogen, Carlsbad, CA, USA) and stored at − 80 °C until RNA isolation.

### Measuring the production of reactive oxygen species

The kidneys were homogenized in ice-cold HEPES buffer (25 mM HEPES, 1 mM EDTA, and 0.1 mM phenyl-methyl-sulfonyl-fluoride). The homogenates were centrifuged at 6000 × *g* for 5 min at 4 °C, and the supernatant was obtained. The oxidation of dihydroethidium (DHE) to ethidium (Eth) was used to measure the production of reactive oxygen species (ROS). The supernatants (10 µl) were incubated with DHE (0.2 mM), salmon teste DNA (10 mg/ml), and the substrate for xanthine oxidase (XO), mitochondrial respiratory enzymes, NADH oxidase, or nitric oxide synthase (NOS). Eth-DNA fluorescence was measured at an excitation wavelength of 480 nm and an emission wavelength of 610 nm at 37 °C for 30 min in a multimode microplate reader (Synergy HT, Biotek, Winooski, VT, USA). Xanthine (0.1 mM) was used as a substrate for XO, and allopurinol (1 mM) was used as an XO inhibitor. Succinate (5 mM) was used as a substrate for mitochondrial ROS production. L-arginine (L-arg, 1 mM) was used as a substrate for NOS, and NADH (0.1 mM) was used as a substrate for NADH oxidase. A blank with no sample was used to measure background fluorescence, and this value was subtracted from each sample reading. Enzyme activity is expressed relative to the control.

### Real time RT-PCR

Total RNA was obtained from kidneys using Trizol, following the manufacturer's instructions. cDNA was synthesized from 2 μg of total RNA under standard reverse transcription conditions, using oligo(dT) in a final volume of 25 μl. cDNA was diluted 1:3, and 2 μl of the diluted sample were used for amplification, using the SYBR Green PCR Master Mix (Life Technologies, Carlsbad, CA, USA) and the following primers: *NOX2*, forward 5’-AGG AGT GCC CAG TAC CAA AGT-3’, reverse 5’-TAC TGT CCC ACC TCC ATC TTG-3’; *NOX4*, forward 5’-ACC CAA GTT CCA AGC TCA TTT-3’, reverse 5’-ATG GTG ACA GGT TTG TTG CTC-3’; *iNOS*, forward 5’-CCA AGC CCT CAC CTA CTT CC-3’, reverse 5’-CTC TGA GGG CTG ACA CAA GG-3’; *eNOS*, forward 5’-CAA CGC TAC CAC GAG GAC ATT-3’, reverse 5’-CTC CTG CAA AGA AAA GCT CTG G-3’; *ICAM-1*, forward 5’-GTG ATG CTC AGG TAT CCA TCC A-3’, reverse 5’-CAC AGT TCT CAA AGC ACA GCG-3’ (Sigma-Aldrich, St. Louis, MO, USA). The expression of the housekeeping gene glyceraldehyde-3-phosphate dehydrogenase (*GAPD*) was also evaluated as an internal control (4352932, Applied Biosystems, Foster City, CA, USA). mRNA levels were calculated from standard curves for each gene, relative to those of GAPD.

### Blood pressure measurement

Systolic and diastolic BP was measured daily within the 11:00–15:00-h interval, to minimize circadian cycle-related variability^59^, under surgical anesthesia (xylazine, 10 mg/kg, i.p.); the animals were kept on a heat pad at 33–34 °C and under a cotton towel to prevent heat loss. Systolic and diastolic blood pressure was measured in mice by a non-invasive method, using a digital plethysmograph tail-cuff placed on the mouse tail; this tail-cuff is connected to a computerized system for data acquisition (LE5002 LETICA®, Panlab, Barcelona, Spain). This equipment closely approximates blood pressure measurement by a sphygmomanometer. The systolic and diastolic BP were measured at the beginning (baseline) and every 5 weeks (on week 0, 5, and 10) until the end of the experiment. Two weeks before the baseline BP measurement and the first AGII application, the mice were subjected to two training sessions, one week apart. Mice with a BP increase of 15% or higher with respect to the baseline (time 0) were considered as hypertensive.

### Histopathology

After the last BP measurement, the mice were anesthetized with sodium pentobarbital (30 mg/kg, i.p.) and perfused with ice-cold PBS (NaCl 140 mM, KCl 2 mM, K_2_HPO_4_ 1.15 mM). The kidneys and livers were removed and fixed in Zamboni’s solution. Then, the tissues were dehydrated and embedded in paraffin. Tissue sections (5 μm) were transferred to poly-L-lysine-coated slides (Sigma) and were deparaffinized and rehydrated. For histopathological studies, the slides were stained with either the Masson trichrome (kidney and liver), hematoxylin-eosin (eyes), or periodic acid-Schiff (PAS) stain (kidney). The Masson trichrome method combines hematoxylin stain with a cytoplasmic stain and a selective stain for connective tissue. The PAS stain, which detects polysaccharides (glycogen and mucosal substances like glycoproteins, glycolipids, and mucins) in tissues to identify alterations in the basement membrane, was used to assess the expansion of the mesangial matrix by the presence of increased amounts of PAS-positive material in the mesangial region^60^. All slides were observed under an ECLIPSE 80i microscope (Nikon, Tokyo, Japan) and analyzed with the software Metamorph v.6.1. (Molecular Devices, San Jose, CA, USA).

Glomerulosclerosis or hyalinization was defined as the disappearance of cellular elements from the tuft, a collapse of capillary lumen, and a folding of the glomerular basement membrane with entrapment of amorphous material, as proposed by Raij et al.^60^. Glomerular injury was also analyzed according to the method proposed by that author, with minor modifications. Briefly, 50–60 cortical glomeruli were evaluated in PAS-stained kidney slides from each group under a 100X objective. Digitized images were analyzed with Metamorph v.6.1. The mesangial area was calculated by subtracting the capillary area from the total area. The results were expressed as percentage with respect to the total area.

Vascular remodeling was evaluated in the portal triad in Masson-stained liver slides. Total and luminal areas of the branch of hepatic artery (BHA) were measured in 10 slides per group under the 40X objective. The area corresponding to vessel thickness was determined by subtracting these two values. Lumen percentage, medial thickness percentage, and media/lumen ratio were calculated to assess vascular remodeling.

### ICAM-1 detection by immunohistochemistry

After deparaffinization and rehydration, tissue sections were incubated with 3% H_2_O_2_ and with 5% albumin with 1% Tween 20-PBS. After treatment, tissue sections were incubated overnight with rat anti-mouse ICAM-1 monoclonal antibody (eBioscence; CAT #: 16-0542) diluted 1:100 in 0.1% albumin, 0.05% Tween 20-PBS. After washing with PBS, the tissues were incubated with a biotinylated goat anti-rat IgG secondary antibody (MP Biomedicals, Santa Ana, CA, USA) diluted 1:1000, followed by HRP-labeled streptavidin solution (MP Biomedicals) at 37 °C for 30 min, and developed with 3,3-DAB (Zymed, San Francisco, CA, USA). All slides were counterstained with hematoxylin–eosin and photographed using an ECLIPSE 80i microscope and analyzed with Metamorph v.6.1.

### Cytokine and sVACM quantification by ELISA

Kidneys were weighed and frozen at − 80 °C until used. The organs were macerated in a frozen mortar with ice-cold PBS-PMSF (0.1%) 1:5 w/v. The suspensions were centrifuged, and supernatants were recovered and frozen at − 20 °C until used. Various ELISA kits were used to determine cytokine concentration, following the manufacturer’s instructions. OptEIA Mouse IL1β, IL4, IL6, IL10, IFNγ, and TNFα ELISA kits were purchased from BD (Franklin Lakes, NJ, USA), while mouse IL17A and TGFβ ELISA kits were purchased from Applied Biosystems (Foster City, CA, USA). sVCAM was provided by Abacam (Waltham, MA, USA). Briefly, 96-well flat-bottomed ELISA plates were coated with the respective capture antibody and incubated overnight at 4 °C. Non-specific binding sites were blocked by incubating for 30 min at RT with PBS-5% fetal bovine serum. Aqueous kidney extracts were added and incubated for 2 h at RT. Then, the plates were incubated with the corresponding detection anti-cytokine-HRP antibodies for 30 min at RT. Bound complexes were detected by reaction with tetramethylbenzidine substrate after 30 min of incubation in the darkness. The reaction was stopped with H_2_SO_4_ 2 N and absorbance was measured at 450 nm at 37 °C in a VERSAmax ELISA plate reader (Molecular Devices). Cytokine and sVACM concentration was calculated according to standard curves for each cytokine and reported as pg/mg protein.

### Statistical analysis

Data were analyzed using the software InStat (GraphPad, San Diego, CA, USA). Data are reported as mean ± standard deviation (SD) and analyzed with Mann–Whitney U-test. Analysis of blood pressure was performed using ANOVA-Tukey test. *P* < 0.05 was considered as statistically significant.

## Supplementary Information

Supplementary Figure S1.

Supplementary Figure S2.

Supplementary Figure S3.

Supplementary Figure S4.
