# Supplementary figures and images for: Characterization of a murine model of endothelial dysfunction induced by chronic intraperitoneal administration of angiotensin II
Source: Sci Rep. 2021 Oct 27;11:21193. doi: 10.1038/s41598-021-00676-x (PMC8551243; doi:10.1038/s41598-021-00676-x)

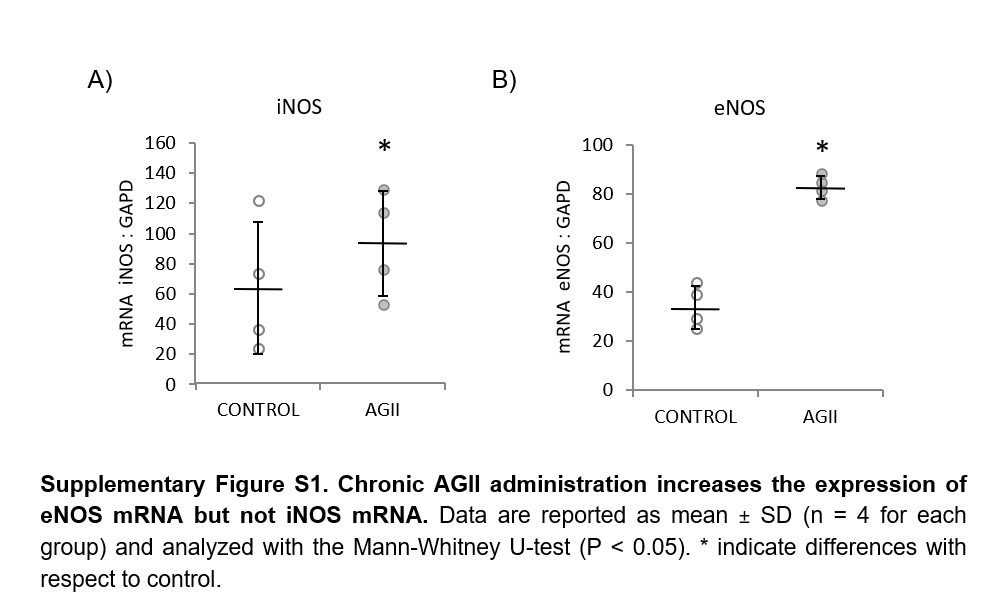

Supplement: Supplementary file 1 — Supplementary Figure S1. [file 41598_2021_676_MOESM1_ESM.tif]

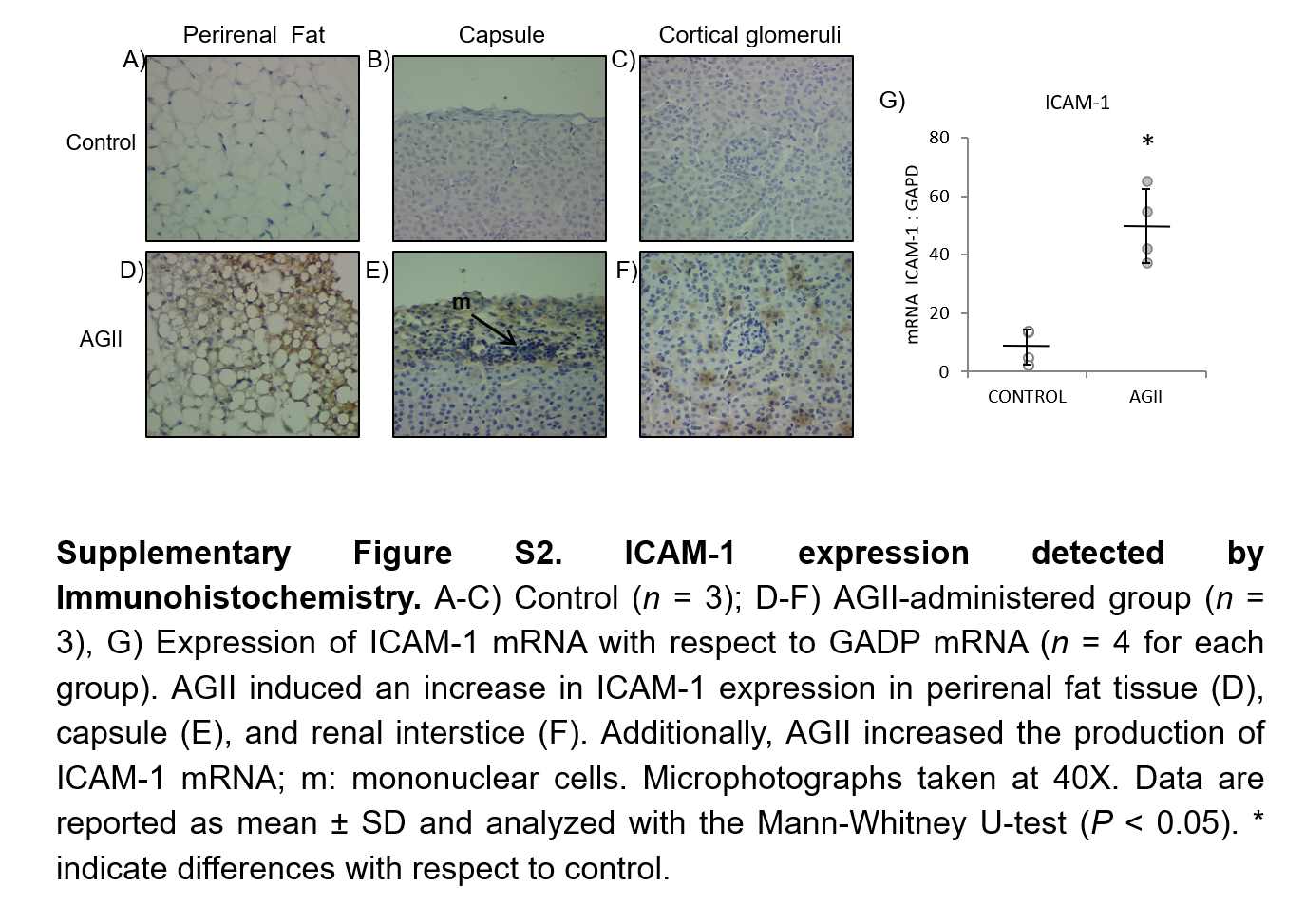

Supplement: Supplementary file 2 — Supplementary Figure S2. [file 41598_2021_676_MOESM2_ESM.tif]

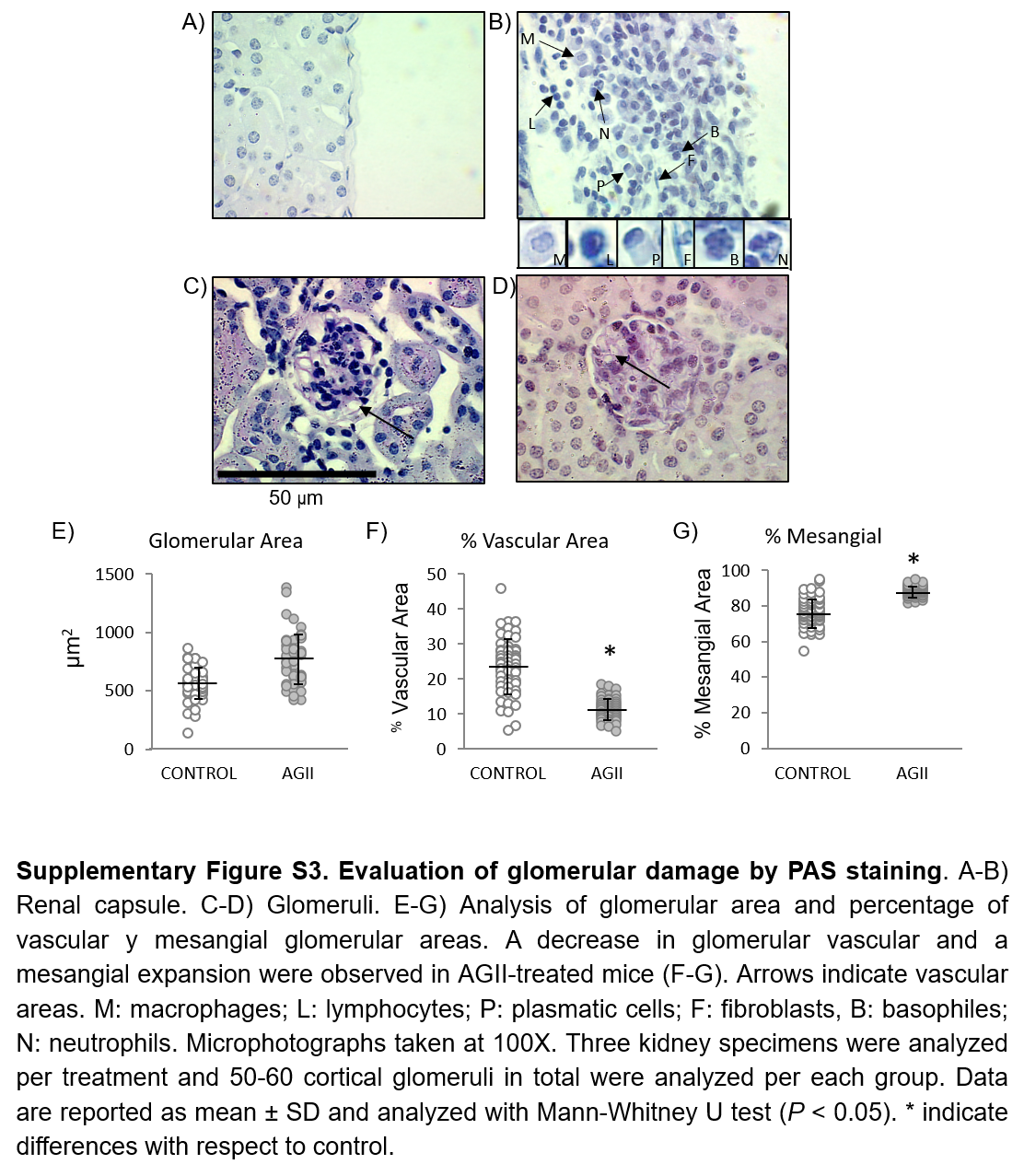

Supplement: Supplementary file 3 — Supplementary Figure S3. [file 41598_2021_676_MOESM3_ESM.tif]

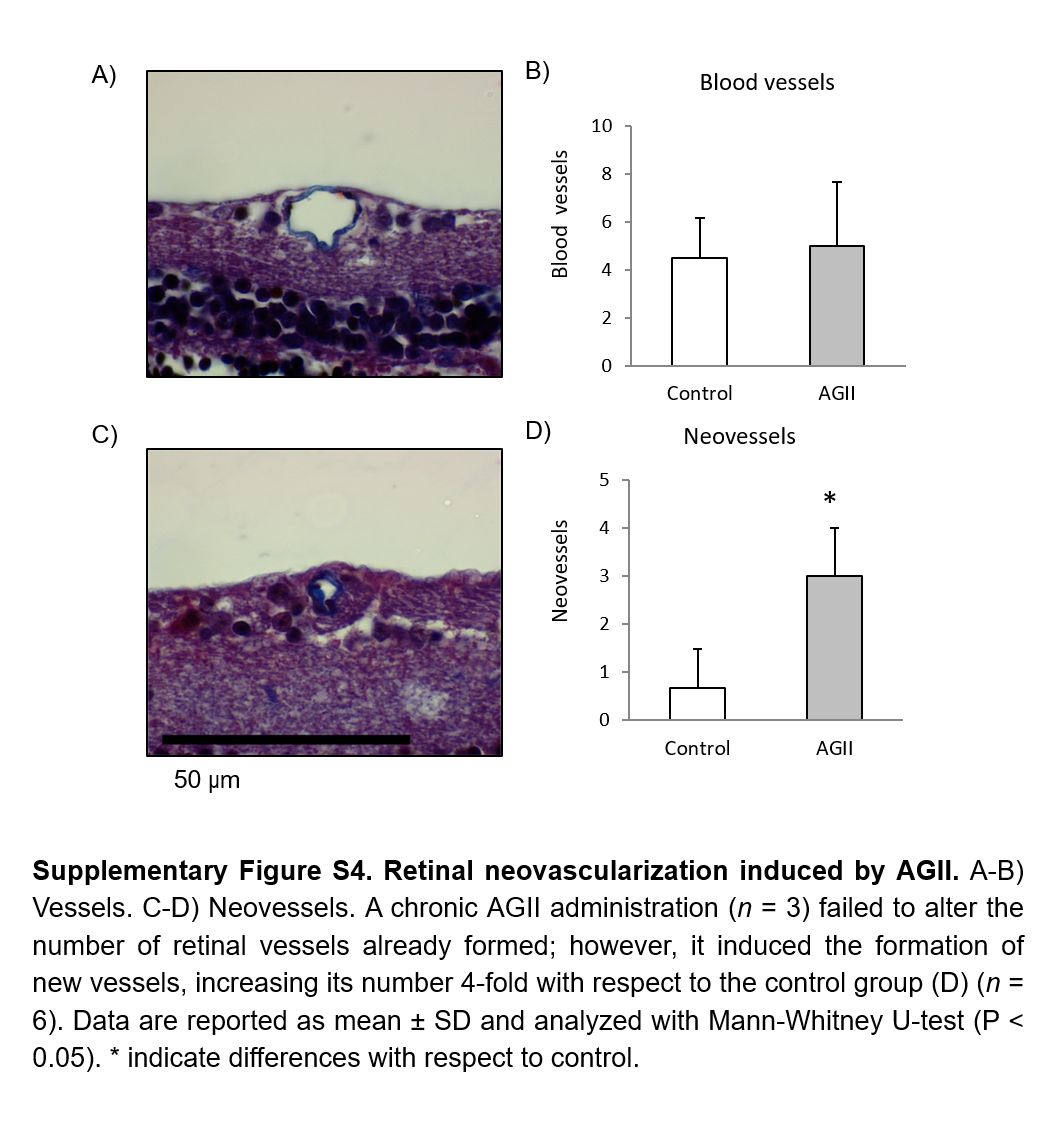

Supplement: Supplementary file 4 — Supplementary Figure S4. [file 41598_2021_676_MOESM4_ESM.tif]
